# Development and Psychometric Evaluation of a Theory-Based Preceptorship Survey for Nurse Practitioners

**DOI:** 10.3390/nursrep15090338

**Published:** 2025-09-17

**Authors:** Leonie DeClerk, Brian Parks

**Affiliations:** College of Nursing, University of Arkansas for Medical Sciences, Little Rock, AR 72205, USA; bjparks@uams.edu

**Keywords:** preceptorship, nurse practitioners, nursing, nursing education, surveys and questionnaires, psychometrics

## Abstract

**Background/Objectives**: Preceptorship is a key aspect of clinical education for healthcare professions, including nurse practitioners (NP). Numerous studies have explored barriers and facilitators to preceptorship; however, few have used a theory-based, psychometrically sound instrument. The aim of this study was to develop and validate an instrument predicting nurse practitioner preceptorship based on the *Integrated Behavioral Model (IBM)*. **Methods**: This was an instrument validation study with a longitudinal design. A pool of 82 statements that reflected constructs of the *IBM* was developed from the existing literature and unpublished studies. Items were evaluated for clarity and construct validity by 20 faculty members and NPs. Further refinement after administration to a small sample of NPs yielded a 60-item Likert-type survey that was sent to NPs in 20 states. A total of 154 NPs repeated the survey after 2–4 weeks to evaluate test–retest reliability. Exploratory and confirmatory factor analysis were used to establish subscales and assess internal consistency, convergent, and discriminant validity. **Results**: 35 items were retained in the final survey. We identified 10 subscales reflecting constructs in the *IBM.* All subscales had adequate internal consistency and discriminant validity. One subscale had inadequate convergent validity and test–retest reliability, while another subscale had inadequate content validity. **Conclusions**: The resultant *Predicting Preceptorship Survey* is theory-based and psychometrically sound. There is no subscale for one *IBM* construct, “salience.” This instrument could be used in studies of engagement in preceptorship in order to identify focus areas for interventions to increase the availability of preceptors, and to evaluate the outcomes of those interventions. Future research should include longitudinal studies of preceptorship and validation of the instrument with other professions, in other countries, and in other cultures.

## 1. Introduction

Preceptorship, partnering students or novice clinicians with expert clinicians, is a common method of education and orientation in healthcare and other professions. However, there is an ongoing shortage of willing and qualified preceptors throughout healthcare [1,2]. This preceptor shortage limits admission to and graduation from healthcare educational programs, including those for nurse practitioners (NPs) [1]. Several recently published studies evaluated barriers and facilitators to preceptorship through cross-sectional survey methodology, e.g., [3,4,5,6,7,8,9,10,11,12,13,14,15]. However, most studies of preceptorship published in the last 25 years have limited or no psychometric evaluation of the instrument, and few use a theoretical or conceptual model to frame the instrument. To effectively address the preceptor shortage, it is crucial to develop a psychometrically sound instrument based on a conceptual framework, which can be used to generate knowledge about preceptorship, modify clinical education strategies, inform interventions focused on preceptors and healthcare organizations, and evaluate the outcomes of those interventions.

### 1.1. Conceptual Frameworks Used in Preceptorship Surveys

Three conceptual frameworks have been used as a basis for studies of preceptorship. Appendix A, Table A1 provides a summary of the studies reviewed.

Kanter’s *Structural Determinants of Behavior in Organizations* [16] remains the most commonly cited conceptual framework in studies of preceptorship [7,9,17,18]. This framework proposes that a worker’s empowerment and commitment to an organization are dependent on opportunities for advancement and access to needed information, support, and resources, as well as formal and informal power. Dibert and Goldenberg integrated this framework into a survey for nursing preceptors [19], which was later adapted for use with graduate nursing preceptors by Donley and associates [18]. The instrument, which the researchers developed from the preceptorship literature to mirror the framework, includes three scales: perceived benefits and rewards of preceptorship, perceived support for preceptorship, and commitment to being a preceptor. However, psychometric evaluation of the instrument and adaptations is limited to face or content validity [8,11,17], internal consistency of scales [7,8,9,17,18,19,20,21,22], and test–retest reliability [18]. Notably, construct validity has not been evaluated for this instrument. Although the survey has been used or adapted for many studies of preceptorship in a variety of healthcare professions, e.g., [4,7,8,9,17,18,20,21], items do not evaluate other factors that might influence the decision to be a preceptor, such as an educational program or the importance of incentives. In addition, the samples have typically included only preceptors, and evaluated commitment to the preceptor role. Therefore, a broader framework that can be used with persons who are not preceptors is needed to evaluate predictors of preceptorship.

Herzberg’s *Dual Factor Theory* proposes that job satisfaction is influenced by motivational and hygiene factors [10]. Motivational factors are internal to the person and increase satisfaction. In the survey by Kauth and Reed [10], motivational factors were reflected in rewards for preceptorship, such as recognition and payment. Hygiene factors are external to the person and decrease satisfaction if they are not present. These factors were reflected as barriers to preceptorship, such as high stress and workload or insufficient time. The instrument has only been evaluated for face and content validity. The *dual factor theory* is narrow and focuses on satisfaction; therefore, a broader framework is needed to capture the multiple factors that will predict engagement in preceptorship.

The *Integrated Behavioral Model* (*IBM*) can be used to predict behavior [23]. In this model *personal factors*, such as socio-demographics, personality, and attitude toward a target, create underlying beliefs [23]. Those beliefs influence the *primary determinants of intent to perform the behavior (primary determinants)* [23]. The *primary determinants* include the following: (1) *attitude toward the behavior*, which includes experiential attitude—how it makes you feel—and instrumental attitude—whether the outcomes are positive or negative; (2) *perceived norm*, which includes injunctive norm—support for the behavior—and descriptive norm—prevalence of the behavior; and (3) *personal agency*, which includes self-efficacy and perceived behavioral control [24]. *Intent to perform the behavior* is the most important predictor of actual behavior; however, *external factors*, such as constraints within the environment, salience of the behavior, habit, and having the prerequisite knowledge and skills, will influence the progression from intent to performance of the behavior [24]. The *IBM* has been used as a framework for other studies of preceptorship [5,25]; however, the authors only report face validity and internal consistency of the survey instrument, have a limited number of items evaluating some aspects of the *IBM*, and do not include subscales reflecting constructs in the *IBM*. A psychometrically sound instrument is needed to effectively use the *IBM* as a framework to generate knowledge about preceptorship, modify clinical education strategies, inform interventions focused on preceptors and healthcare organizations, and evaluate the outcomes of those interventions.

### 1.2. IBM Constructs in Studies of Preceptorship

*Personal factors* include socio-demographic characteristics, as well as personality and attitude toward students [25]. Previous studies indicate that years of experience in the professional role and hours worked per week influence engagement in preceptorship [5,26,27,28]. Personality is not supported as a predictor of preceptorship [5]. Attitude toward students has not been evaluated, although positive student characteristics are discussed as facilitators of preceptorship [4,25,29].

*Primary determinants of behavioral intent* include experiential and instrumental attitude, injunctive and descriptive norms, self-efficacy, and perceived behavioral control [24]. Experiential attitudes described in the literature include enjoyment, satisfaction, pride, fulfillment, fatigue, stress, and feeling drained [3,4,5,6,7,8,9,10,12,28,30,31]; however, no experiential attitude is predictive of preceptorship. Instrumental attitudes that are predictive of engagement in preceptorship include perception of the preceptor as a role model and appreciation of preceptorship [5]. Other instrumental attitudes in the literature include recognition, prestige, professional advancement, and keeping up to date [4,5,10,12,13,27,32]. In relation to injunctive norms, perceived support from patients, colleagues, supervisors and administrators, and collaborating physicians are predictive of preceptorship status [5], and lack of support is described as a barrier to preceptorship [25,27,29]. Considering descriptive norms, knowing other NPs who are preceptors is a predictor of engagement in preceptorship, but agreement that preceptorship is a professional obligation is not [5]. Self-efficacy is typically evaluated as having sufficient expertise clinically and in teaching, being confident, and having adequate preparation [5,6,15,22,25,28,30]. Elements of behavioral control that have been evaluated include access to technology, credentialing requirements, and scheduling, as well as more generic statements related to the ease of being a preceptor in the setting [4,5,10,13].

*External factors*, including environmental constraints, salience, habit, program factors, the value placed on incentives, maker of the preceptorship decision, and frequency of requests, have been evaluated in several studies. The most commonly described environmental constraint is having sufficient time [3,4,5,6,7,8,9,10,12,15,25,26,28,29,32]; however, although one study found that having protected time increased capacity to engage in preceptorship [26], another found that having sufficient time was not a predictor of engagement in preceptorship [5]. Other environmental constraints include specialty, suitable patients, suitable setting, perceived workload, productivity requirements, space, electronic medical record, and risk for liability [3,4,5,15,25,28,32]. Salience has only been evaluated in two studies [5,30]; however, lower agreement that it was important the NP be a preceptor was a predictor of status as a former, rather than current, preceptor [5]. Data on habit, frequency of being a preceptor, has not been evaluated as a predictor of preceptorship [5]. Knowledge and skills, i.e., having received training to be a preceptor, do not predict participation in preceptorship [5,10,26]. Based on previous research that indicated that many preceptors had not received training, knowledge and skills were removed from our model [5,33,34]. Factors related to the educational program that were evaluated in previous studies and which support engagement in preceptorship include clear responsibilities, communication, availability of faculty, on-site faculty visits, available resources, previous relationship with the program, and the particular program that students attend [3,5,6,7,8,9,10,12,25,26,28,31,32]. Incentives, such as payments and tax credits, continuing education, awards and honors, and tokens of appreciation are useful, but do not appear to be a major factor in the decision to be a preceptor [3,5,10,11,12,13,15,25,27,29]. The person making the preceptorship decision and number of requests to be a preceptor are predictive of preceptorship status [5].

### 1.3. Purpose of This Study

Having an available supply of preceptors is imperative for NP education. However, few studies of engagement in preceptorship use a survey instrument based on a conceptual framework with extensive psychometric evaluation. Only one framework used, the *IBM*, is a predictive framework with engagement in the behavior as an outcome. One recent study used a survey based on the *IBM* to predict NPs’ engagement in preceptorship; however, the survey underwent limited psychometric evaluation [5]. To our knowledge, there are no validated *IBM*-based instruments for evaluating preceptorship in NPs. However, the development of a psychometrically sound instrument, framed within a robust predictive conceptual model, is essential for research purposes. Furthermore, knowledge gained from studies using such an instrument could inform educational practices, guide curriculum development, and enhance the quality of preceptorship. Therefore, the aim of this study was to develop and validate an instrument predicting nurse practitioner preceptorship based on the *IBM*.

## 2. Materials and Methods

### 2.1. Study Design

This was an instrument validation study with a longitudinal design. Standard steps were followed for survey development [35]. The initial steps included (1) generation of item pool, (2) assessment of content validity, (3) cognitive interviewing to evaluate clarity of items, and (4) subscale refinement through a developmental sample. The survey was then deployed to a final sample, with EFA and confirmatory factor analysis of final results used to further refine subscales and establish internal consistency, as well as convergent and discriminant validity. Test–retest reliability was also evaluated using volunteers from the final sample.

### 2.2. Ethical Considerations

The protocol for survey development was submitted to the Institutional Review Board (IRB) of the University of Arkansas for Medical Sciences (UAMS). Initial survey development was determined not to be human subjects research (protocol 276010, 22 June 2023). Final survey deployment and analysis were approved as exempt (protocol 276866, 15 February 2024). Consent information was provided on the landing page of the surveys for both the developmental sample and the final sample. Participation was voluntary and no personal information was required. However, participants who requested a USD 25 gift card for completing cognitive interviewing or a USD 10 gift card for completing the survey, and those who volunteered to participate in the test–retest evaluation or a future study, were required to provide their first and last name, email address, license number, and state of licensure. All data were collected in Research Electronic Data Capture (REDCap^®^), and results were downloaded as Excel spreadsheets and stored in the principal investigator’s (PI) UAMS Box account. REDCap^®^ is a secure, web-based program that can be used to create surveys and other database projects [36]. Names and email addresses of participants volunteering to provide retest data or for a future study were retained as a separate spreadsheet. Gift card receipts were downloaded as a separate spreadsheet and stored in a separate folder in the PI’s UAMS Box account. All personal identifiers were deleted from the database in REDCap^®^, as well as from the file used for data analysis. The list of retest volunteers was permanently deleted after data collection was completed. The PI completed all data analysis and is the only person with access to identifiable data.

### 2.3. Item Generation

An 82-item pool was generated, based on the existing literature and unpublished findings, using the *IBM* as a theoretical model. Statements were developed to reflect various constructs in the *IBM*, including subscales for attitude toward students (12 items), experiential attitude (10 items), instrumental attitude (10 items), injunctive norm (6 items), descriptive norm (5 items), self-efficacy (4 items), behavioral control (6 items), salience (5 items), environmental constraints (9 items), incentives (9 items), and program factors (6 items). The statements were entered into REDCap^®^ to be rated using a 6-point Likert-type scale from “strongly disagree” to “strongly agree”. Appendix B, Table A2 shows the statements in the initial survey and the stage at which they were removed, if applicable.

### 2.4. Content Validity

To establish content validity, the statements were reviewed by eight NP faculty members with experience with preceptors. Reviewers were provided with a summary of the *IBM*, which served as the conceptual framework, and were asked to rate the statements as “applicable”, “probably applicable”, or “not applicable” in relation to an NP’s decision to be a preceptor. Content validity index for individual items (I-CVI) was calculated by dividing the number of reviewers who rated an item as “applicable” or “probably applicable” by the total number of reviewers for the item. Although nine items had an I-CVI < 0.80, they were retained because they had a basis in published or unpublished research. After final subscales were determined, the average CVI for each subscale (S-CVI/Ave) was calculated by summing the I-CVI of each subscale item and dividing by the number of items in that subscale. CVI for the instrument was calculated by summing the I-CVI for all items in the instrument and dividing by the total number of items.

### 2.5. Cognitive Interviewing

Twelve NPs completed cognitive interviewing to refine the survey’s clarity and further evaluate content validity. During cognitive interviewing, the NP completed the survey while the investigator watched over Zoom. The investigator asked clarifying questions to elicit how the NP interpreted the questions, as well as whether any questions were unclear or not applicable to preceptorship. During this time, 14 items were edited to specify NP students and five items were edited to improve clarity. One item from the instrumental attitude subscale, which had inconsistent interpretation by the NPs and was not able to be clarified, was removed after cognitive interviewing.

### 2.6. Initial Subscale Refinement

To further refine the survey, it was disseminated through email to contacts who were known NPs, and through snowball sampling from those contacts. This developmental sample included 125 NPs. We used scale reliability analysis in IBM SPSS^®^ version 29 software to decrease the number of items and to improve the internal consistency of each subscale. When two items were similar, e.g., “being a preceptor makes me anxious” and “being a preceptor makes me nervous,” the one with lower variability was removed. The resultant instrument included 60 items thought to reflect the 11 constructs associated with the *IBM*. After receiving approval from UAMS IRB, this instrument was deployed to the final sample.

### 2.7. Final Sample Recruitment

Eligible participants were NPs who were residents of the United States, had practiced as an NP for at least two years, and were currently practicing in a paid or volunteer position. Names and postal or email addresses were identified from lists downloaded or purchased from 18 state boards of nursing or other licensing bodies. Invitations to complete the survey were sent by postcard or email to up to 3000 NPs in each state, although nine states had fewer than 3000 NPs. Email addresses of NPs in two other states (Texas and Florida) were identified from the 18 state lists. In total, 46,417 invitations were sent. Demographic information was also collected. Five times the number of items is adequate for exploratory factor analysis (EFA), and ten to twenty times the number of items is considered sufficient for confirmatory factor analysis (CFA) [37]. With 60 items on the survey, the target recruitment was at least 600 participants.

### 2.8. Data Handling and Management of Missing Data

Data were downloaded from REDCap^®^ as an Excel spreadsheet and uploaded into IBM SPSS^®^ version 29 for analysis. Ineligible responses—those who were not NPs, did not practice in the United States, or had not been in practice for two years, as well as one response that did not provide any data—were removed at that time. Responses to questions that were worded negatively, or which were expected to receive negative responses, were reverse coded. The data were separated into two approximately equal files using the random file selection feature in IBM SPSS^®^ version 29. One data file was used for EFA and the second for CFA. There was less than 1.1% missing data for any individual item, which is not expected to change outcomes [38]. For EFA, entries with missing data were omitted listwise. For CFA, missing data were imputed using the regression imputation feature of IBM AMOS^®^ version 26.

### 2.9. Exploratory and Confirmatory Factor Analysis

The Kaiser–Meyer–Olkin Measure of Sampling Adequacy (KMO) and Bartlett’s Test of Sphericity were evaluated to ensure that the sample was suitable for factor analysis. EFA, using principal component analysis and varimax rotation with Kaizer normalization, was used to identify subscales. We removed items with multiple cross-loadings. Scale reduction techniques were used to improve the internal consistency of subscales by removing additional items.

CFA was completed using structural equation modeling in IBM AMOS^®^ version 26 in order to evaluate convergent validity (average variance extracted [AVE]) and discriminant validity (maximum shared variance [MSV] and average shared variance [ASV]). Additional items were removed during CFA to improve convergent and discriminant validity. Model fit indices were also evaluated.

We repeated EFA with retained items and removed additional items that had multiple cross-loadings, then repeated CFA to evaluate convergent and discriminant validity of the final subscales.

The initial EFA identified twelve factors, ranging from two to six items. Thirteen items were removed during the EFA because of multiple cross-loadings. During scale improvement following the initial EFA, one item was removed to improve the internal consistency of the NP program subscale, and two items that formed another subscale were removed due to poor internal consistency. During the initial CFA, four subscales were noted to have inadequate convergent validity (AVE < 0.5), and the item with the lowest standardized regression weight was removed from each subscale. All subscales demonstrated adequate discriminant validity. During the second EFA, one subscale evidenced multiple cross-loadings. The four items were removed sequentially; however, all items continued to have cross-loadings, and the subscale, which contained items expected to reflect positive attitudes towards preceptorship and salience, was removed. During the second EFA, two subscales were noted to have inadequate convergent validity. One item was removed from one subscale, which resulted in adequate convergent validity. It was not possible to improve convergent validity for the second subscale. We repeated EFA and CFA on the final instrument using the separated files. Those results are reported below.

### 2.10. Internal Consistency

Subscales were named to reflect the items and were quantified by summing individual scores for each item of that subscale. We evaluated the internal consistency of the final subscales in IBM SPSS^®^ version 29 using the entire dataset.

### 2.11. Test–Retest Reliability

To evaluate test–retest reliability (temporal stability), the survey instrument was emailed after two weeks to a sample of 346 volunteers who provided an email address and indicated their willingness to repeat the survey at two weeks. To enable matching of these responses with the participants’ initial responses, each participant was provided with the line number of the original response to use as an identifier. At least 100 responses are considered sufficient to establish test–retest reliability and internal consistency of a scale [39]. Responses for initial and second surveys were matched by respondent number and uploaded in IBM SPSS^®^ version 29. Items in each subscale were totaled for the initial and second surveys, and then compared through intraclass correlation coefficient (ICC) using a two-way mixed-effects model and an absolute agreement definition [40]. Results were reported using ICC for single measures and 95% confidence interval.

## 3. Results

Only 1295 valid responses were received from the 46,417 postcards or emails sent for the full survey (2.8% response rate). A total of 154 responses were received from the 346 emails sent for the retest survey (44.5% response rate). Participants were 88% female, 87.7% white, and 6.4% Hispanic; 58.1% worked in an urban setting, and 56.3% were certified as family nurse practitioners (see Table 1 for demographics). The average age of participants was 46.25 years (*SD* = 10.72) for the full sample and 46.26 years (*SD* = 11.55) for the retest sample.

### 3.1. Consistency of Final Scale with Conceptual Framework

After instrument refinement, 10 subscales were identified that included 2 to 5 items each, 35 items in total. Two items reflecting negative experiential attitudes toward preceptorship loaded to a single factor. However, no items expected to reflect positive attitude toward preceptorship or salience loaded to a factor without multiple cross-loadings. Other items loaded to factors that reflected constructs in the *IBM*. Table 2 delineates the construct, subscale name, and number of items.

### 3.2. Content Validity of Final Subscales

CVI for the instrument was 0.948. Nine subscales had S-CVI/Ave over 0.800, reflecting adequate content validity. One subscale, *expectation*, had S-CVI/Ave of 0.667. All items in this subscale had I-CVI < 0.80 when rated by NP faculty members during scale development. The S-CVI/Ave for each subscale is presented in Table 3a,b.

### 3.3. EFA for Final Subscale

The 10 subscales explained 74.94% of the variance in the sample. KMO and Bartlett’s Test were adequate throughout the instrument refinement; KMO = 0.848 and Bartlett’s Test Χ^2^ ≈ 12,228.118, *df* = 595, *p* < 0.001 in factor analysis of the items included in the final instrument. See Table 3a,b for rotated component matrix, with values < 0.35 excluded, and Table A3 for the complete rotated component matrix.

### 3.4. CFA for Final Subscale

Model fit indices for the final CFA included Root Mean Square Error of Approximation (RMSEA) = 0.054 (90% CI; 0.050, 0.057), Comparative Fit Index (CFI) = 0.928, and Tucker–Lewis Index (TLI) = 0.917. Convergent validity was adequate for nine subscales (AVE > 0.5) [41]. AVE for the *personal benefit* subscale was 0.421, indicating inadequate convergent validity. Discriminant validity was adequate for all subscales, with ASV and MSV < AVE [41]. AVE, ASV, and MSV for each subscale are presented in Table 3a,b.

### 3.5. Internal Consistency of Final Subscales

All subscales had adequate internal consistency, with α ≥ 0.70 [35]. Three subscales had excellent internal consistency (α ≥ 0.90), two were good (0.9 > α ≥ 0.8), and five were adequate (0.8 > α ≥ 0.7). The Cronbach’s alpha for each subscale is presented in Table 3a,b.

### 3.6. Test–Retest Validity of Final Subscales

Nine subscales had adequate test–retest reliability with α ≥ 0.70 [35]; α = 0.635 for the *personal benefit* subscale. Other authors have recommended α of 0.5–0.75 indicating moderate reliability [42]. The ICC for each subscale is presented in Table 3a,b.

## 4. Discussion

The 35-item *Predicting Preceptorship Survey* was based on the *IBM*, a robust behavioral model. The 10 subscales reflected constructs in the *IBM*. The *attitude toward students* subscale reflected attitude toward the target in the *IBM*. Experiential and instrumental attitude were reflected in the *negative attitude toward preceptorship* and *personal benefit* subscales. Injunctive and descriptive norms were reflected in the *support* and *expectation* subscales. Self-efficacy and perceived control were reflected in the *self-efficacy* and *behavioral control* subscales. In relation to external factors, environmental constraints, program factors, and incentives were reflected in the *setting*, *NP program*, and *incentives* subscales. Items expected to reflect positive attitudes toward preceptorship and salience were deleted during EFA, and no subscales were developed for those constructs.

Unlike other surveys used to study preceptorship, this instrument was based on a robust conceptual framework and underwent extensive psychometric evaluation, including content, convergent, and divergent validity, as well as internal consistency of subscales and test–retest reliability. The most commonly used survey to study preceptorship in nursing is based on Kanter’s *Structural Determinants of Behavior in Organizations* framework [16]; however, there is limited psychometric evaluation beyond internal consistency. One study reported an adequate CVI of an adapted version of two of the three subscales [22]; two other researchers reported face validity [8,17], and one researcher reported test–retest reliability of their adaptation of the survey, with α = 0.65 [18]. In addition, the outcome variable of this survey was commitment to the preceptorship role, not engagement in preceptorship. Items were phrased in a way that precluded use with participants who are not preceptors, and there was no evaluation of construct validity of the subscales. One survey instrument, based on Herzberg’s *Dual Factor Theory*, underwent face and content validity evaluation through faculty review and piloting with local NPs [10]; however, the outcome variable of this instrument was satisfaction, not behavior. The *IBM* was used as a conceptual framework for one previous cross-sectional survey study [5]. Psychometric evaluation was limited to face validity, as well as internal consistency of two subscales that were thought to reflect primary determinants of the decision to be a preceptor as well as external factors [5]. However, the *IBM* is a superior conceptual framework because it predicts engagement in a behavior.

This study produced a validated, *IBM*-based survey that can be used for studies of NP preceptorship. The survey has some limitations. The AVE of one subscale, *personal benefit*, was 0.421, and therefore did not meet the threshold for convergent validity. In addition, the *personal benefit* subscale had ICC with α < 0.70; however, all met the less stringent requirement recommended by Koo and Li for establishing adequate test–retest reliability [42]. Another subscale, *expectation*, did not meet the threshold for content validity for individual items or scale average. Overall, this survey is an effective option for predicting and evaluating NPs’ engagement in preceptorship. The *personal benefit* subscale could be revised and re-evaluated in future studies.

### 4.1. Limitations

There were limitations with this psychometric evaluation of the *Predicting Preceptorship Survey*. The response rate for the full survey was low at 2.8%, creating possible selection bias. NPs who responded may differ from those who did not. The participants were primarily female and white, and most were certified as family NPs. Although this is consistent with NPs in the United States, other populations may differ from these participants. It is not possible to evaluate non-responders. The survey was sent through postcards and emails, so it is not known whether the recruitment materials reached all the NPs. Some non-responders emailed and stated they were not eligible, were retired, or that the monetary incentive was not sufficient; however, the reasons for not participating are unknown for most non-responders. The low response rate and homogeneity of the sample may limit generalizability to other populations.

Three aspects of validity—criterion, cross-cultural, and ecological—were not evaluated during this study. We did not locate a similar validated instrument that could be compared for criterion validity. The instrument did not meet Consensus-based Standards for the selection of health Measurement Instruments (COSMIN) criteria for structural validity that require RMSEA < 0.06 and CFI or TLI > 0.95, as well as the first factor of the EFA accounting for at least 20% of variance [43]. In this study, RMSEA was <0.06; however, CFI and TLI were <0.95, and the first factor in the EFA, *support*, only accounted for 12% of variance. In addition, this instrument was only evaluated with practicing NPs in the United States, limiting generalizability to other professions, countries, or cultures.

Other variables—such as age, years of experience, control over the preceptorship decision, and time available for preceptorship—that are supported as important in the preceptorship decision, were not included in the original instrument or were removed from the instrument during scale refinement [5,25,26,31,33]. These items should be included as individual items in future studies of preceptorship. In addition, researchers could consider including a validated personality instrument, which was not included in this survey due to the large number of items. In the *IBM*, these individual items would fall under the *personal factors* or *external factors* categories. The individual items were not included in instrument development because we were most interested in developing validated subscales; however, the omission of these constructs may affect the sensitivity of the instrument. This could be evaluated in future studies.

### 4.2. Indications for Practice and Education

This instrument can be used in studies of NPs’ engagement in preceptorship. Having a better understanding of why NPs do not engage in preceptorship is crucial to finding effective interventions to increase the supply of willing and available NP preceptors. This survey could be used in a pre–post format to evaluate the effectiveness of interventions. Knowledge gained from studies using this survey would have the potential to change the way that preceptors are recruited and retained by NP programs. In addition, there is a potential to identify aspects external to the NP, such as support for preceptorship in the organization, importance of program interactions, and incentives that can provide actionable insights into the preceptor shortage.

### 4.3. Indication for Future Research

This instrument was developed and evaluated with NPs in the United States. Another theory-based survey has been adapted and used with nurse preceptors in other countries [19]; however, the current survey is more robust and suitable for non-preceptors as well as preceptors. Researchers should evaluate this instrument for use in other countries and with other professions. For adaptation, it would be imperative to evaluate content validity, because this survey was developed for a specific nursing role and country. Nurses and NPs in other countries and members of other professions may have different responsibilities and work environments. In addition, care must be taken with translation and adaptation to address any cultural or linguistic differences and to ensure applicability to the target population.

This instrument can be used to evaluate the efficacy of the *IBM* as a conceptual framework for addressing preceptorship. Previous studies of preceptorship have been cross-sectional; a longitudinal study, evaluating the same participants over time, could provide evidence of the relevance of this model in clinical education. The *IBM* is a predictive model, with the intent to perform a behavior being acted on by external factors to inhibit or allow that behavior. We plan to repeat the survey study with volunteers from the initial sample to establish whether responses from the initial study do predict the movement from preceptorship intent to preceptorship behavior after two years. Understanding whether and how the various constructs in this model, which are operationalized in this survey, predict engagement in preceptorship will form a foundation for future educational interventions to increase the availability of preceptors.

## 5. Conclusions

The *Predicting Preceptorship Survey* is a psychometrically sound instrument for evaluating *IBM* constructs in relationship to NP preceptorship. The instrument includes 10 subscales, reflecting most constructs of the *IBM*. We evaluated content, convergent, and discriminant validity, as well as internal consistency and test–retest reliability. One subscale, *personal benefit*, had inadequate convergent validity and test–retest reliability. A second scale, *expectation*, had inadequate content validity. Subscales for salience and positive attitude toward preceptorship were not identified. This instrument could be used in studies of engagement in preceptorship, to identify focus areas for interventions to increase the availability of preceptors, and to evaluate the outcomes of those interventions. Future research should include longitudinal studies of preceptorship and validation of the instrument with other professions, in other countries, and in other cultures.

## Figures and Tables

**Table 1:** Demographic profile of participants.

Variable	Full Sample (*N* = 1295)		Retest Sample (*N* = 154)	
	*n*	%	*n*	%
Preceptorship Status				
Current (within 2 years)	775	59.8	96	62.3
Former (>2 years ago)	299	23.1	30	19.5
Never	221	17.1	28	18.2
Gender				
Female	1139	88.0	139	90.3
Male	134	10.3	13	8.4
Other/Unanswered	22	1.7	2	1.3
Race				
White	1136	87.7	141	91.6
Black/African American	74	5.7	4	2.6
Asian	49	3.8	6	3.9
Other/Unanswered	36	2.8	3	1.9
Specialty Certification ^1^				
Family NP	729	56.3	97	63
Adult/Adult Gerontology Primary Care or Gerontology NP	255	19.7	23	14.9
Adult/Adult Gerontology Acute Care NP	209	16.1	22	14.3
Psychiatric–Mental Health NP	152	11.7	15	9.7
Pediatric Acute/Primary Care NP	120	9.3	8	5.2
Women’s Health NP	63	4.9	9	5.8
Neonatal NP	21	1.6	1	0.6
Practice Location				
Rural (<2500)	138	10.7	19	12.3
Small Urban (2500–49,999)	352	27.3	36	23.4
Urban (≥50,000)	749	581	95	61.7
Telehealth Only	50	3.9	4	2.6

^1^ Specialty certifications total > 100% because some participants had multiple certifications.

**Table 2:** Construct, subscale name, and number of items in final instrument.

Construct	Subscale	Items
Attitude toward students	Attitude toward students	4
Experiential attitude	Negative attitude toward preceptorship	2
Instrumental attitude	Personal benefit	3
Injunctive norm	Support	5
Descriptive norm	Expectation	3
Perceived control	Behavioral control	4
Self-efficacy	Self-efficacy	4
Salience	N/A	
Environmental constraints	Setting	3
Program factors	NP program	3
Incentives	Incentives	4

**Table 3:** (**a**) Rotated component matrix and psychometric evaluation for first five subscales. (**b**) Rotated component matrix and psychometric evaluation for remaining subscales.

(**a**)					
**Abbreviated Statement**	**Support**	**Self-Efficacy**	**Attitude Toward Students**	**Behavioral Control**	**NP Program**
Supportive supervisors	0.926				
Supportive organization	0.911				
Supportive NPs and physicians	0.860				
Preceptorship encouraged	0.826				
Supportive nurses and staff	0.768				
Confident in ability to precept		0.905			
Sufficient experience as an NP		0.894			
Enough experience as a teacher/mentor		0.891			
Adequate preparation to precept		0.875			
Students understand foundational concepts			0.862		
Students well-prepared			0.766		
Students read in preparation			0.749		
Students eager to learn			0.740		
Access to technology				0.798	
Access to necessary resources				0.759	
Guide student through steps				0.733	
Overcome barriers				0.623	
Communication with faculty					0.898
Faculty easily available					0.886
Clear expectations of preceptor					0.852
Cronbach’s alpha	0.935	0.934	0.805	0.839	0.920
AVE	0.760	0.773	0.502	0.585	0.840
ASV	0.131	0.056	0.015	0.161	0.027
MSV	0.521	0.229	0.039	0.521	0.161
S-CVI/Ave	1.000	1.000	0.969	0.969	1.000
Test–retest (95% CI)	0.742 (0.642, 0.818)	0.808 (0.728, 0.866)	0.813 (0.736, 0.869)	0.717 (0.608, 0.799)	0.724 (0.619, 0.803)
(**b**)					
**Abbreviated Statement**	**Incentives**	**Expectation**	**Setting**	**Personal Benefit**	**Negative Attitude Toward Preceptorship**
Tax credit	0.816				
Payment	0.804				
Discounts	0.708				
Free continuing education	0.688				
Most NPs are preceptors		0.888			
Most NPs I know are preceptors		0.882			
Preceptorship is expected by most employers		0.698			
Enough patients			0.815		
Suitable population and specialty			0.809		
Underlying atmosphere appropriate			0.739		
Helps NPs advance professionally				0.848	
Role models				0.813	
Make a difference				0.673	
Preceptorship draining					0.897
Preceptorship stressful					0.828
Cronbach’s alpha	0.775	0.783	0.789	0.706	0.785
AVE	0.501	0.578	0.531	0.421	0.754
ASV	0.023	0.040	0.114	0.006	0.028
MSV	0.161	0.190	0.441	0.014	0.094
S-CVI/Ave	0.959	0.667	0.952	0.952	0.988
Test–retest	0.825 (0.725, 0.878)	0.746 (0.626, 0.828)	0.822 (0.747, 0.877)	0.635 (0.503, 0.738)	0.719 (0.605, 0.802)

AVE = average variance extracted, ASV = average shared variance, MSV = maximum shared variance, S-CVI/Ave = average content validity index for subscale, 95% CI = 95% confidence interval.

## Data Availability

The final, anonymized dataset is not publicly available, as permission was not obtained from participants to share data.

## Public Involvement Statement

There was no public involvement in any aspect of this research.

## Guidelines and Standards Statement

This manuscript was drafted against the Recommendations for reporting the results of studies of instrument and scale development testing [44].

## Abbreviations

The following abbreviations are used in this manuscript:

NP	Nurse practitioner
CVI	Content validity index
*IBM*	Integrated Behavioral Model
REDCap^®^	Research Electronic Data Capture
EFA	Exploratory factor analysis
CFA	Confirmatory factor analysis
KMO	Kaiser–Meyer–Olkin Measure of Sampling Adequacy
AVE	Average variance extracted
MSV	Maximum shared variance
ASV	Average shared variance
ICC	Intraclass correlation coefficient
RMSEA	Root Mean Square Error of Approximation
CFI	Comparative Fit Index
TLI	Tucker–Lewis Index

## Appendix A

This appendix shows a summary of preceptorship studies using conceptual frameworks and reporting psychometric evaluation of the instrument reviewed in this article.

**Table A1:** Summary of literature.

Author	Development	Identified Framework	Validity	Reliability	Profession
Adapted from survey by Dibert & Goldenberg [19]					
Hykäs & Shoemaker [20]	Adapted verbiage			Internal consistency	Nurses
Chang et al. [21]	Adapted verbiage			Internal consistency	Nurses
Cloete & Jeggels [17]	Adapted verbiage	Kanter	Face	Internal consistency	Nurses
Wang et al. [22]	Adapted		Content	Internal consistency	Nurses
Macey et al. [7]	Adapted	Kanter		Internal consistency	Nurses
Alhassan et al. [8]	Adapted, new subscale		Face	Internal consistency	Nurses
Gholizedah et al. [9]	Adapted and translated	Kanter		Internal consistency	Nurses
Donley et al. [18]	Adapted	Kanter		Internal consistency Test–retest	Multiple
Other surveys with identified conceptual framework					
DeClerk et al. [5]	Adapted surveys and existing literature	*Integrated behavioral model*	Face	Internal consistency	Nurse practitioners
Kauth & Reed [10]	Researcher developed Existing literature	*Dual factor theory*	Face Content		Nurses

## Appendix B

### Appendix B.1

This appendix shows the constructs of the *IBM*, the statements that were used to evaluate each construct, and the stage of survey development at which they were removed from the instrument, if applicable. All statements were rated on a 6-point Likert-type scale from “strongly disagree” to “strongly agree.” Negative statements were reverse coded prior to analysis.

**Table A2:** Constructs, subscale name, statements, content validity index, and stage removed, if not included in final instrument.

Construct	Statement (Code for Retained Items)	I-CVI	Stage Removed ^1^
Attitude toward students	Most NP students are well-prepared. (ATS1)	1	
	Most NP students are eager to learn. (ATS2)	1	
	Most NP students understand foundational concepts. (ATS3)	1	
(Attitude toward students)	I am afraid that I will get a bad student.	0.875	DS
	Most NP students are motivated.	1	DS
	Most NP students behave professionally.	1	CFA1
	NP students often arrive late or leave early.	1	DS
	Most NP students read in preparation for clinical. (ATS5)	0.875	
	Most NP students are able to complete an appropriate history and physical examination.	1	DS
	Most NP students can develop differential diagnoses.	1	DS
	Most NP students understand appropriate medications to prescribe for common conditions I see.	1	DS
	Most NP students are not interested in my patients.	0.625	DS
Experiential attitude	Being a preceptor is enjoyable.	1	EFA2
	Being a preceptor is exciting.	1	EFA2
(Negative attitude toward preceptorship)	Being a preceptor is satisfying.	1	EFA2
	Being a preceptor is draining. (EA4R)	0.875	
	Being a preceptor makes me nervous.	0.875	EFA1
	Being a preceptor is stressful. (EA6R)	1	
	Being a preceptor fills me with dread.	0.75	DS
	I am (would be) proud to be a preceptor.	1	EFA2
	I am (would be) honored to be a preceptor.	1	DS
	Being a preceptor makes me anxious.	0.875	DS
Instrumental attitude	Preceptors are role models. (IA1)	1	
	Preceptorship increases nurse practitioners’ esteem.	0.75	DS
(Personal benefit)	Preceptorship helps nurse practitioners advance professionally. (IA3)	0.857	
	There are no benefits to being a preceptor.	1	EFA1
	Preceptors are appreciated.	1	EFA1
	Preceptors make a difference. (IA6)	1	
	Preceptors give back to the profession.	1	DS
	Preceptorship interferes with your relationship with your patients.	0.875	DS
	Preceptorship takes a lot of time.	1	DS
	Preceptorship is inconvenient.	0.75	CI
Injunctive norm	My organization is supportive of preceptorship. (DN1)	1	
	My supervisors & administrators are supportive of preceptorship. (DN2)	1	
(Support)	The NPs & physicians I work with are supportive of preceptorship. (DN3)	1	
	The nurses & other staff I work with are supportive of preceptorship. (DN4)	1	
	Preceptorship is encouraged in my organization. (DN5)	1	
	Preceptorship is rewarded in my organization.	1	EFA1
Descriptive norm	Most nurse practitioners I know are preceptors for NP students. (DN1)	0.625	
	Most nurse practitioners are preceptors for NP students. (DN2)	0.75	
(Expectation)	Preceptorship is a professional responsibility.	0.875	SI
	Preceptorship is an expectation of nurse practitioners.	1	SI
	Preceptorship is expected by most employers. (DN5)	0.625	
Self-efficacy	I have sufficient expertise as a nurse practitioner to be a preceptor. (SE1)	1	
	I am confident in my ability to be a preceptor. (SE2)	1	
(Self-efficacy)	I have enough experience as a teacher or mentor to be a preceptor. (SE3)	1	
	I have adequate preparation to be a preceptor. (SE4)	1	
Perceived control	I can easily overcome barriers to be a preceptor. (BC1)	1	
	It is difficult to rearrange my schedule to be a preceptor.	1	CFA1
(Behavioral control)	I can easily access any resources I need to be a preceptor. (BC3)	0.875	
	I can guide a student through the steps to be precepted in my setting. (BC4)	1	
	I can easily provide the student with access to the technology needed for me to be their preceptor. (BC5)	1	
	It is difficult to be a preceptor in my setting.	1	EFA1
Salience	It is important that I am a preceptor.	1	EFA1
	Preceptorship is important for NP education.	1	CFA1
(N/A)	Preceptorship is important for my professional growth.	1	EFA1
	Preceptorship is important for my recertification.	1	EFA1
	Preceptorship is an expectation of my employment.	0.75	DS
Environmental constraints	I have sufficient time to be a preceptor.	1	EFA1
	I have sufficient space to be a preceptor.	1	EFA1
	My patient population and clinical specialty are suitable for nurse practitioner students. (EC3)	1	
(Setting)	I have enough patients to meet the needs of a student. (EC4)	1	
	The underlying atmosphere of my practice setting is appropriate for students. (EC5)	0.857	
	I am concerned about students’ reactions to my patients.	0.75	DS
	I am concerned about my patients’ reactions to students.	1	DS
	I am concerned about legal liability when I am a preceptor.	1	EFA1
Incentives	Having access to online clinical resources would make me more likely to be a preceptor.	0.875	EFA1
(Incentives)	Having library privileges would make me more likely to be a preceptor.	0.875	DS
	Having adjunct faculty status would make me more likely to be a preceptor. (The “adjunct faculty” title does not include payment or additional responsibilities.)	1	CFA1
	Receiving a tax credit would make me more likely to be a preceptor. (INC3)	1	
	Receiving payment would make me more likely to be a preceptor. (INC4)	1	
	Receiving discounts for university events would make me more likely to be a preceptor. (INC5)	0.875	
	Receiving free continuing education would make me more likely to be a preceptor. (INC6)	1	
	Receiving a thank-you letter would make me more likely to be a preceptor.	1	EFA1
	Receiving an award would make me more likely to be a preceptor.	1	CFA2
Program factors	Knowing faculty are easily available if I have concerns about a student would make me more likely to be a preceptor. (PF1)	1	
(NP program)	Knowing how to communicate with the nurse practitioner program would make me more likely to be a preceptor. (PF2)	1	
	Having clear expectations of my responsibilities would make me more likely to be a preceptor. (PF3)	1	
	Knowing faculty will be present for site visits would make me more likely to be a preceptor.	0.875	SI
	Understanding the responsibilities of the faculty in relation to my role would make me more likely to be a preceptor.	1	DS
	Understanding the responsibilities of the faculty in relation to the student would make me more likely to be a preceptor.	1	DS

^1^ Note: CI indicates removed after cognitive interviewing, DS indicates removed after survey completed by developmental sample, EFA1 indicates removed during first EFA, SI indicates removed following EFA1 to improve subscale, CFA1 indicates removed during first CFA to improve convergent or discriminant validity, EFA2 indicates removed during second EFA, CFA2 indicates removed during second CFA.

### Appendix B.2

This appendix shows the rotated component matrix with all values. Due to page width, original codes for items are listed. Abbreviated statements are provided in Table 3a,b.

**Table A3:** Rotated component matrix for final factor analysis.

Component										
	1	2	3	4	5	6	7	8	9	10
IN2	0.926	0.056	0.070	0.136	0.018	0.052	0.092	0.088	0.039	0.025
IN1	0.911	0.045	0.037	0.154	0.011	0.068	0.078	0.065	0.082	0.009
IN3	0.860	0.097	0.104	0.104	0.027	0.071	0.067	0.148	0.054	0.062
IN5	0.826	0.017	0.060	0.150	0.127	0.039	0.212	0.029	0.044	−0.013
IN4	0.768	0.127	0.118	0.221	−0.012	0.056	0.080	0.187	0.150	0.082
SE2	0.078	0.905	0.053	0.171	0.019	−0.003	−0.050	0.151	0.118	0.031
SE3	0.084	0.894	0.023	0.189	0.059	0.032	−0.030	0.091	0.081	0.032
SE1	0.048	0.891	0.040	0.092	−0.016	−0.010	−0.044	0.160	0.072	0.009
SE4	0.091	0.875	0.026	0.242	−0.015	0.011	−0.009	0.064	0.074	0.057
ATS3	0.061	−0.001	0.862	0.047	0.005	−0.005	0.007	0.042	0.101	0.066
ATS1	0.156	0.009	0.766	0.034	0.072	−0.015	0.088	0.121	0.088	0.074
ATS5	0.048	0.032	0.749	0.092	0.062	0.004	0.161	−0.085	0.083	0.102
ATS2	0.043	0.073	0.740	0.001	0.073	0.002	0.025	0.099	0.135	0.014
BC5	0.175	0.140	0.136	0.798	0.081	0.028	0.059	0.066	0.050	0.055
BC3	0.164	0.248	−0.036	0.759	0.090	−0.050	0.050	0.107	0.117	0.060
BC4	0.212	0.258	0.039	0.733	0.065	0.071	0.050	0.163	0.090	0.065
BC1	0.308	0.162	0.081	0.623	0.109	0.113	−0.017	0.244	0.023	0.179
PF2	0.042	0.033	0.038	0.057	0.898	0.161	0.040	0.073	0.055	−0.060
PF1	0.056	0.016	0.092	0.098	0.886	0.119	0.063	0.037	0.049	−0.034
PF3	0.032	−0.007	0.090	0.094	0.852	0.117	0.031	0.044	0.088	0.017
INC3	0.017	0.024	0.022	−0.011	0.031	0.816	0.016	0.093	0.028	−0.198
INC4	0.040	0.002	0.011	−0.096	−0.026	0.804	−0.067	0.049	0.003	−0.192
INC5	0.070	0.003	−0.063	0.106	0.226	0.708	0.104	−0.020	−0.014	0.159
INC6	0.143	−0.002	0.012	0.144	0.287	0.688	0.062	0.050	−0.008	0.088
DN1	0.118	−0.044	0.109	0.041	0.035	0.019	0.888	0.111	0.081	0.035
DN2	0.096	0.001	0.169	0.078	−0.007	0.056	0.882	0.105	0.024	0.072
DN5	0.228	−0.079	0.006	0.004	0.114	0.020	0.698	−0.102	−0.017	−0.143
EC4	0.030	0.169	0.060	0.165	0.036	0.038	0.012	0.815	0.008	0.031
EC3	0.156	0.170	0.073	0.088	0.073	0.063	0.069	0.809	0.087	0.030
EC5	0.342	0.102	0.062	0.198	0.059	0.083	0.044	0.739	0.086	0.017
IA3	0.077	0.042	0.108	0.120	0.053	0.021	0.052	0.015	0.848	0.061
IA1	0.133	0.131	0.080	0.061	0.052	−0.053	−0.018	0.069	0.813	−0.009
IA6	0.071	0.142	0.285	0.039	0.093	0.050	0.058	0.078	0.673	0.083
EA4R	0.039	−0.030	0.126	0.056	−0.017	−0.085	0.009	0.035	0.025	0.897
EA6R	0.084	0.152	0.129	0.194	−0.057	−0.079	−0.044	0.039	0.106	0.828

Extraction method: principal component analysis. Rotation method: varimax with Kaizer normalization. Rotation converged in six iterations.

### Appendix B.3

This appendix shows the standardized regression weights of items on each factor (Table A4), and covariances of each factor (Table A5) for final CFA.

**Table A4:** Standardized regression weights.

			Estimate
IN5	<---	IN	0.825
IN4	<---	IN	0.788
IN3	<---	IN	0.851
IN2	<---	IN	0.952
IN1	<---	IN	0.931
SE4	<---	SE	0.837
SE3	<---	SE	0.881
SE2	<---	SE	0.921
SE1	<---	SE	0.876
PF3	<---	PF	0.869
PF2	<---	PF	0.970
PF1	<---	PF	0.908
ATS5	<---	ATS	0.665
ATS3	<---	ATS	0.789
ATS2	<---	ATS	0.658
ATS1	<---	ATS	0.713
BC5	<---	BC	0.778
BC4	<---	BC	0.844
BC3	<---	BC	0.704
BC1	<---	BC	0.725
EC3	<---	EC	0.749
EC4	<---	EC	0.625
EC5	<---	EC	0.800
DN1	<---	DN	0.886
DN2	<---	DN	0.856
DN5	<---	DN	0.467
INC3	<---	INC	0.896
INC4	<---	INC	0.783
INC5	<---	INC	0.507
INC6	<---	INC	0.574
IA1	<---	IA	0.755
IA3	<---	IA	0.552
IA6	<---	IA	0.623
EA4R	<---	EAR	0.605
EA6R	<---	EAR	1.069

**Table A5:** Covariances.

			Estimate	S.E.	C.R.	P
IN	<-->	SE	0.214	0.047	4.596	***
IN	<-->	PF	0.051	0.053	0.945	0.345
IN	<-->	ATS	0.171	0.043	4.000	***
IN	<-->	BC	0.722	0.071	10.127	***
IN	<-->	INC	0.009	0.070	0.133	0.895
IN	<-->	IA	0.063	0.023	2.716	0.007
IN	<-->	EC	0.598	0.065	9.236	***
IN	<-->	DN	0.436	0.070	6.268	***
EAR	<-->	IN	0.167	0.046	3.659	***
SE	<-->	PF	0.135	0.040	3.379	***
SE	<-->	ATS	0.128	0.031	4.060	***
SE	<-->	BC	0.479	0.051	9.448	***
SE	<-->	INC	−0.003	0.051	−0.049	0.961
SE	<-->	IA	0.089	0.018	5.065	***
SE	<-->	EC	0.334	0.044	7.514	***
SE	<-->	DN	0.173	0.049	3.530	***
EAR	<-->	SE	0.221	0.043	5.166	***
PF	<-->	ATS	0.032	0.036	0.886	0.376
PF	<-->	BC	0.177	0.052	3.403	***
PF	<-->	INC	0.401	0.064	6.273	***
PF	<-->	IA	0.079	0.020	3.882	***
PF	<-->	EC	0.061	0.047	1.294	0.196
PF	<-->	DN	0.150	0.058	2.598	0.009
EAR	<-->	PF	−0.011	0.034	−0.311	0.756
ATS	<-->	BC	0.197	0.042	4.748	***
ATS	<-->	INC	−0.030	0.047	−0.631	0.528
ATS	<-->	IA	0.112	0.017	6.454	***
ATS	<-->	EC	0.146	0.038	3.871	***
ATS	<-->	DN	0.095	0.044	2.136	0.033
EAR	<-->	ATS	0.082	0.029	2.849	0.004
BC	<-->	INC	−0.004	0.067	−0.059	0.953
BC	<-->	IA	0.118	0.023	5.144	***
BC	<-->	EC	0.664	0.066	10.076	***
BC	<-->	DN	0.286	0.065	4.430	***
EAR	<-->	BC	0.306	0.058	5.293	***
INC	<-->	IA	−0.018	0.026	−0.686	0.493
EC	<-->	INC	0.087	0.062	1.412	0.158
DN	<-->	INC	0.055	0.075	0.739	0.460
EAR	<-->	INC	−0.181	0.051	−3.534	***
EC	<-->	IA	0104	0.021	4.896	***
DN	<-->	IA	0.010	0.024	0.416	0.677
EAR	<-->	IA	0.035	0.015	2.297	0.022
EC	<-->	DN	0.164	0.059	2.803	0.005
EAR	<-->	EC	0.209	0.046	4.549	***
EAR	<-->	DN	−0.023	0.042	−0.555	0.579

*** indicates *p* < 0.001.

## References

[1] American Association of Colleges of Nursing & American Organization for Nursing Leadership (2020). AACN-AONL Clinical Preceptor Survey Summary Report.

[2] Alfonso S., Lund M., Moore M., Leppke A., Vohra-Khullar P. (2023). Significant primary care preceptor shortage remains one year after the pandemic started. Ann. Fam. Med..

[3] Boyce D.J., Shifrin M.M., Moses S.R., Moss C.R. (2022). Perceptions of motivating factors and barriers to precepting. J. Am. Assoc. Nurse Pract..

[4] Clarke C.L., Larson S., Feret B., Dy-Boarman E., Abu-Baker A. (2024). Adjunct preceptor perceptions of motivation, understanding, and support for the precepting role. Am. J. Pharm. Educ..

[5] DeClerk L., Lefler L., Nagel C., Mitchell A., Sparbel K. (2022). Why don’t all nurse practitioners precept? A comparative study. J. Am. Assoc. Nurse Pract..

[6] Regaira-Martínez E., Ferraz-Torres M., Mateo-Cervera A.M., Vázquez-Calatayud M. (2023). Nurses’ perceptions of preceptorship of undergraduate students in clinical context. J. Prof. Nurs..

[7] Macey A., Green C., Jarden R.J. (2021). ICU nurse preceptors’ perceptions of benefits, rewards, supports and commitment to the preceptor role: A mixed-methods study. Nurse Educ. Pract..

[8] Alhassan A., Fuseini A.G., Osman W., Dadinkai I.A., Mahama S.S. (2022). Preceptors’ perceptions of support, commitment to the preceptor role, and preferred incentives: A cross-sectional study. Nurse Educ. Today.

[9] Gholizadeh L., Shahbazi S., Valizadeh S., Mohammadzad M., Ghahramanian A., Shohani M. (2022). Nurse preceptors’ perceptions of benefits, rewards, support, and commitment to the preceptor role in a new preceptorship program. BMC Med. Educ..

[10] Kauth C., Reed J.M. (2024). Nurse preceptor motivations, barriers, and perceived rewards post-pandemic. MEDSURG Nurs..

[11] Wang Y., Hu S., Yao J., Pan Y., Wang J., Wang H. (2024). Clinical nursing mentors’ motivation, attitude, and practice for mentoring and factors associated with them. BMC Nurs..

[12] Wiggins H., Martinez R., Franklin H., Perry C.K. (2025). Nurse practitioner preceptors’ perceived facilitators and barriers to precepting. J. Am. Assoc. Nurse Pract..

[13] Delgado F., Immaneni S., MacKelfresh J.B., Yeung H. (2023). Teaching motivators, facilitators, and barriers among dermatology volunteer clinical faculty. Arch. Dermatol. Res..

[14] Golić Jelić A., Tasić L., Odalović M., Stoisavljević Šatara S., Stojaković N., Marinković V. (2020). Predictors and motivation of preceptors’ interest in precepting of pharmacy interns—Do we have a useful questionnaire?. J. Contin. Educ. Health Prof..

[15] Renda S., Fingerhood M., Kverno K., Slater T., Gleason K., Goodwin M. (2022). What motivates our practice colleagues to precept the next generation?. J. Nurse Pract..

[16] Kanter R.M. (1993). Men and Women of the Corporation.

[17] Cloete I.S., Jeggels J. (2014). Exploring nurse preceptors’ perceptions of benefits and support of and commitment to the preceptor role in the Western Cape Province. Curationis.

[18] Donley R., Flaherty M.J., Sarsfield E., Burkhard A., O’Brien S., Anderson K.M. (2014). Graduate clinical nurse preceptors: Implications for improved intra-professional collaboration. Online J. Issues Nurs..

[19] Dibert C., Goldenberg D. (1995). Preceptors’ perceptions of benefits, rewards, supports and commitment to the preceptor role. J. Adv. Nurs..

[20] Hyrkäs K., Shoemaker M. (2007). Changes in the preceptor role: Re-visiting preceptors’ perceptions of benefits, rewards, support and commitment to the role. J. Adv. Nurs..

[21] Chang A., Douglas M., Breen-Reid K., Gueorguieva V., Fleming-Carroll B. (2013). Preceptors’ perceptions of their role in a pediatric acute care setting. J. Contin. Educ. Nurs..

[22] Wang W.F., Hung C.H., Li C.Y. (2018). Development Trajectories and Predictors of the Role Commitment of Nursing Preceptors. J. Nurs. Res..

[23] Fishbein M., Yzer M.C. (2003). Using theory to design effective health behavior interventions. Commun. Theory.

[24] Montaño D., Kaspryzk D., Glantz K., Rimer B., Viswanath K. (2015). Theory of reasoned action, theory of planned behavior, and the integrated behavioral model. Health Behaviors: Theory, Research and Practice.

[25] DeClerk L., Chasteen S., Wells C., Baxter J., Rojo M. (2024). To precept or not to precept: Perspectives from nurse practitioners. J. Am. Assoc. Nurse Pract..

[26] Abey S., Lea S., Callaghan L., Shaw S., Cotton D. (2015). Identifying factors which enhance capacity to engage in clinical education among podiatry practitioners: An action research project. J. Foot Ankle Res..

[27] Gonzalez-Colaso R., Moloney-Johns A., Sivahop J. (2013). To teach or not to teach: 2011 national survey of physician assistants and preceptor experiences. J. Physician Assist. Educ..

[28] Newstead C., Johnston C., Wakely L., Nisbet G. (2024). An exploration of factors influencing physiotherapists’ involvement in student clinical education. Physiother. Theory Pract..

[29] Badeeb N., Alsolami Y., Alhamrani M., Hassanin F., Aalam W. (2025). Exploring motivators and challenges for preceptors to teach in the clinical settings: A survey-based study. BMC Med. Educ..

[30] Knutsen J.S., Bondevik G.T., Hunskaar S. (2025). General practitioners’ attitudes and motivation to supervise medical students in clinical placements: A questionnaire study from Norway. Scand. J. Prim. Health Care.

[31] Todd B.A., Brom H., Blunt E., Dillon P., Doherty C., Drayton-Brooks S., Hung I., Montgomery K., Peoples L., Powell M. (2019). Precepting nurse practitioner students in the graduate nurse education demonstration: A cross-sectional analysis of the preceptor experience. J. Am. Assoc. Nurse Pract..

[32] Cervera-Gasch A., Macia-Soler L., Torres-Manrique B., Mena-Tudela D., Salas-Medina P., Orts-Cortes M.I., González-Chordaá V. (2017). Questionnaire to measure the participation of nursing professionals in mentoring students. Investig. Educ. Enferm..

[33] Alexandraki I., Kern A., Beck Dallaghan G.L., Baker R., Seegmiller J. (2024). Motivators and barriers for rural community preceptors in teaching: A qualitative study. Med. Educ..

[34] Lofgren M., Dunn H., Dirks M., Reyes J. (2021). Perspectives, experiences, and opinions precepting advanced practice registered nurse students. Nurs. Outlook.

[35] DeVellis R.F. (2017). Scale Development: Theory and Applications.

[36] Patridge E.F., Bardyn T.P. (2018). Research Electronic Data Capture (REDCap). J. Med. Libr. Assoc..

[37] Kyriazos T.A. (2018). Applied psychometrics: Sample size and sample power considerations in factor analysis (EFA, CFA) and SEM in general. Psychology.

[38] Fowler F.J. (2014). Survey Research Methods.

[39] Kennedy I. (2022). Sample size determination in test-retest and Cronbach alpha reliability estimates. Br. J. Contemp. Educ..

[40] McGraw K.O., Wong S.P. (1996). Forming inferences about some intraclass correlation coefficients. Psychol. Methods.

[41] Cheung G.W., Cooper-Thomas H.D., Lau R.S., Wang L.C. (2024). Reporting reliability, convergent and discriminant validity with structural equation modeling: A review and best-practice recommendations. Asia Pac. J. Manag..

[42] Koo T.K., Li M.Y. (2016). A guideline of selecting and reporting intraclass correlation coefficients for reliability research. J. Chiropr. Med..

[43] Prinsen C.A., Vohrra S., Rose M.R., Boers M., Tugwell P., Clarke M., Williamson P.R., Terwee C.B. (2016). Guidelines for Selecting Outcome Measurement Instruments for Outcomes Included in a Core Outcome Set.

[44] Streiner D.L., Kottner J. (2014). Recommendations for reporting the results of studies of instrument and scale development and testing. J. Adv. Nurs..

